# Clinicopathological study of pseudomyogenic hemangioendothelioma

**DOI:** 10.1186/s13000-023-01309-9

**Published:** 2023-02-20

**Authors:** Ningning Yang, Yuchen Huang, Panpan Yang, Wentian Yan, Shan Zhang, Nan Li, Zhenzhong Feng

**Affiliations:** 1 The Second Affiliated Hospital of Anhui Medical University, No.678 Furong Road, Hefei, 230000 China; 2 Lu ‘an People’s Hospital, Jindu Garden South District, Lu’an, 237000 China; 3 The Second People’s Hospital of Hefei, No.246 Heping Road, Hefei, 230000 China

**Keywords:** Pseudomyogenic hemangioendothelioma, Clinicopathological features, Immunohistochemistry, Molecular pathology

## Abstract

**Objectives:**

Pseudomyogenic hemangioendothelioma (PHE) is a rare intermediate hemangioendothelioma. This article aims to study the clinicopathological features of PHE.

**Methods:**

We collected the clinicopathological features of 10 new PHE, and examined their molecular pathological features by fluorescence in situ hybridization. In addition, we summarized and analyzed the pathological data of 189 reported cases.

**Results:**

The case group consisted of six men and four women aged 12–83 years (median: 41 years). Five instances occurred in the limbs, three in the head and neck, and two in the trunk. Tumor tissues were composed of spindle cells and round or polygonal epithelioid cells, which were either arranged in sheets or interwoven, along with areas of transitional morphology. Scattered or patchy stromal neutrophil infiltration was observed. Tumor cells had abundant cytoplasm, and some contained vacuoles. The nuclei had mild to moderate atypia, with visible nucleoli, and mitosis was rare. PHE tissues diffusely expressed CD31 and ERG, but not CD34, Desmin, SOX-10, HHV8 or S100, while some samples expressed CKpan, FLI-1 and EMA. INI-1 stain is retained. The proliferation index of Ki-67 is 10–35%. Seven samples were detected by fluorescence in situ hybridization, six of which had breakages in FosB proto-oncogene (AP-1 transcription factor subunit). Two patients experienced recurrence; however, no metastasis or death occurred.

**Conclusions:**

PHE is a rare soft tissue vascular tumor, which has biologically borderline malignant potential, local recurrence, little metastasis, and good overall survival and prognosis. Immunomarkers and molecular detection are valuable for diagnosis.

**Supplementary Information:**

The online version contains supplementary material available at 10.1186/s13000-023-01309-9.

## Introduction

Pseudomyogenic hemangioendothelioma (PHE) is a newly identified intermediate soft tissue tumor. Although the name was first proposed by Hornick et al*.* [1] in 2011, the disease has been discussed for many years. In 1992, Mirra et al*.* [2] first reported five tumors that were histologically similar to epithelioid sarcoma (ES) but lacked its typical characteristics, and called them “variants of epithelioid sarcoma.” In 2003, Billings et al*.* [3] reported seven patients with low-grade vascular tumors similar to ES, and put forward the concept of “Epithelioid sarcoma-like hemangioendothelioma” In 2011, Hornick et al*.* [1], in the largest PHE study to date, reported 50 new cases, further expanding our understanding of these tumors; they named the condition “pseudomyogenic vascular endothelial tumors” to emphasize their histological muscle-like characteristics. Although the name remains controversial [4], the World Health Organization (WHO) officially classified PHE as an intermediate type of soft tissue vascular tumor in 2013 [5].

PHE is rare. It generally occurs in the limbs of young men, especially in the distal lower limbs, but can also occur in various other tissues, including the oral cavity [6, 7], chest wall [1, 8, 9], breast [10], esophagus [11] and external genitalia [1, 12, 13]. It can involve multiple tissue planes, including the dermis, subcutaneous tissue, bone, and skeletal muscle. It is often multifocal and rarely metastasizes; however, it is prone to local recurrence. In this study, we report 10 cases of PHE and comprehensively summarize the details of previously reported cases, to improve our understanding of PHE.

## Materials and methods

### Case data and specimen collection

Tissue specimens and clinical data were collected from 10 patients with PHE, including three cases from the First Affiliated Hospital of Bengbu Medical College (Bengbu, China), three from Shanghai Ruijin Hospital (Shanghai, China), two from the First Affiliated Hospital of Anhui Medical University (Hefei, China), one from the First Affiliated Hospital of the University of Science and Technology of China (Hefei, China), and one from Yijishan Hospital (Wuhu, China). All the published literature in PubMed (https://www.ncbi.nlm.nih.gov/pubmed/) was comprehensively searched, using the keywords “pseudomyogenic hemangioendothelioma” or “epithelioid sarcomatoid hemangioendothelioma,” and repeated case reports were excluded to obtain a non-redundant dataset of cases. In total, there were 189 cases of PHE reported in 59 studies (Supplementary Table 1).

### Pathological examination

Hematoxylin–eosin staining reagent was purchased from Beijing Zhongshan Jinqiao Co., Ltd. (Beijing, China). Antibodies were purchased from Fuzhou Maixin Biotechnology Development Co., Ltd. (Fuzhou, China) (Table 1). Immunohistochemical staining was performed by EnVision's two-step method. The main immunohistochemical markers were CKpan, CD34, CD31, FLI-1, EMA, INI-1, S100, SOX-10, HHV8, Desmin and Ki-67. Two pathologists re-evaluated the sections.

**Table 1:** Antibodies used in immunohistochemistry and their sources

Antibody	Clone number	Monoclonal/Polyclonal	Positive site
CK (pan)	MX005	monoclonal	cytoplasm
CD34	QBEnd/10	monoclonal	cell membrane and cytoplasm
CD31	JC/70A	monoclonal	cell membrane
FLI-1	MX045	monoclonal	nucleus
ERG	MXR004	monoclonal	nucleus
INI-1	MRQ-27	monoclonal	nucleus
S100	4C4.9	monoclonal	cytoplasm/nucleus
SOX-10	EP268	monoclonal	nucleus
Desmin	D33	monoclonal	cytoplasm
Vimentin	V9	monoclonal	cytoplasm
EMA	E29	monoclonal	cytoplasm/cell membrane
HHV8	13B10	monoclonal	nucleus

### Fluorescence in situ hybridization

FOSB (19q13) gene fragment probe reagent, in situ hybridization blue staining solution and related reagents required for in situ hybridization experiment were purchased from Ambiping Pharmaceutical Technology Co., Ltd. (Guangzhou, China). According to the instructions, 7 PHE cases were analyzed by fluorescence in situ hybridization.

### Data collection and analysis

Positive immunohistochemical signals for FLI-1, INI-1, HHV8 and Ki-67 were located in the nucleus, while those for CKpan,and Desmin were observed in the cytoplasm. CD31 was observed in the cell membrane, and CD34 was observed in the cell membrane or cytoplasm. S100 and ERG signals were observed in the nucleus or cytoplasm. Immunohistochemical staining results were scored according to their intensity and range (the percentage of total cancer cells that were stained). The scoring for staining intensity was as follows: colorless, 0 points; pale yellow, 1 point; light brown, 2 points; dark brown, 3 points. The staining range was scored as follows: < 5%, 0 points; 5–25%, 1 point; 26–50%, 2 points; 51–75%, 3 points; > 75%, 4 points. The two scores were then multiplied, and samples scoring 0 points were considered negative; 1–4 points, weakly positive; 5–8 points, moderately positive; 9–12 points, strongly positive. Fluorescence in situ hybridization signals in cancer cell nuclei were observed and quantified under a high-power microscope. A red signal indicated > 10% separation between the number of red and green signals in 50 cells, meaning that the FosB proto-oncogene, AP-1 transcription factor subunit (*FOSB*) gene, was broken and rearranged; with < 10% separation, samples were considered negative.

### Patient follow-up

The patients were followed up for 2–55 months through outpatient services and telephone calls. Follow-up data on the 189 previously reported cases were summarized.

## Results

### Clinical data

The patients included six males and four females, aged 12–83 years, with a median age of 41 years (Table 2). Three cases had the tumor in the lower limbs, three in the head and neck, two in the upper limbs, and two in the trunk. The masses of seven patients were painless, while three patients were treated for painful nodules. Multiple tumors were observed in 5/10 cases. Approximately 25% of PHE cases have bone involvement [1], and among the 10 patients in this group, one was treated for bone tumors in the left knee and one for bone tumors in the left foot. Among the 189 PHE cases screened, 139 were male and 50 were female. Their ages were widely distributed, ranging from 6 to 86 years, with an average age of 34 years. The disease occurred in the limbs, trunk, head, and face, but was concentrated in the limbs (140 cases, 74%), especially in the legs (112 cases, 59%). It was also occasionally detected in the oral cavity, vulva, and breast and often showed multifocal involvement (134 cases, 71%).

**Table 2:** Clinical data from 10 patients

Patient number	Sex	Age	Site	Single/ multiple nodules	Recurrence/ metastasis	Treatment	Follow-up (months)
1	Female	51	neck	Mul	Recurrence	E	3
2	Male	21	forearm	Sin	No	E	2
3	Female	22	foot	Mul	Recurrence	E	55
4	Female	83	Face	Sin	No	E	13
5	Male	12	femur	Mul	Recurrence	E	18
6	Male	22	back	Mul	Recurrence	E + C	31
7	Male	32	arm	Mul	No	E	12
8	Female	63	knee	Sin	No	E	51
9	Male	73	neck	Sin	No	E	22
10	Male	70	neck	Sin	No	E	18

*Abbreviations: E* Excision, *C* Chemotherapy, *Mul* Multiple, *Sin* Single

### Histopathology and immunohistochemistry

**Fig. 1:**
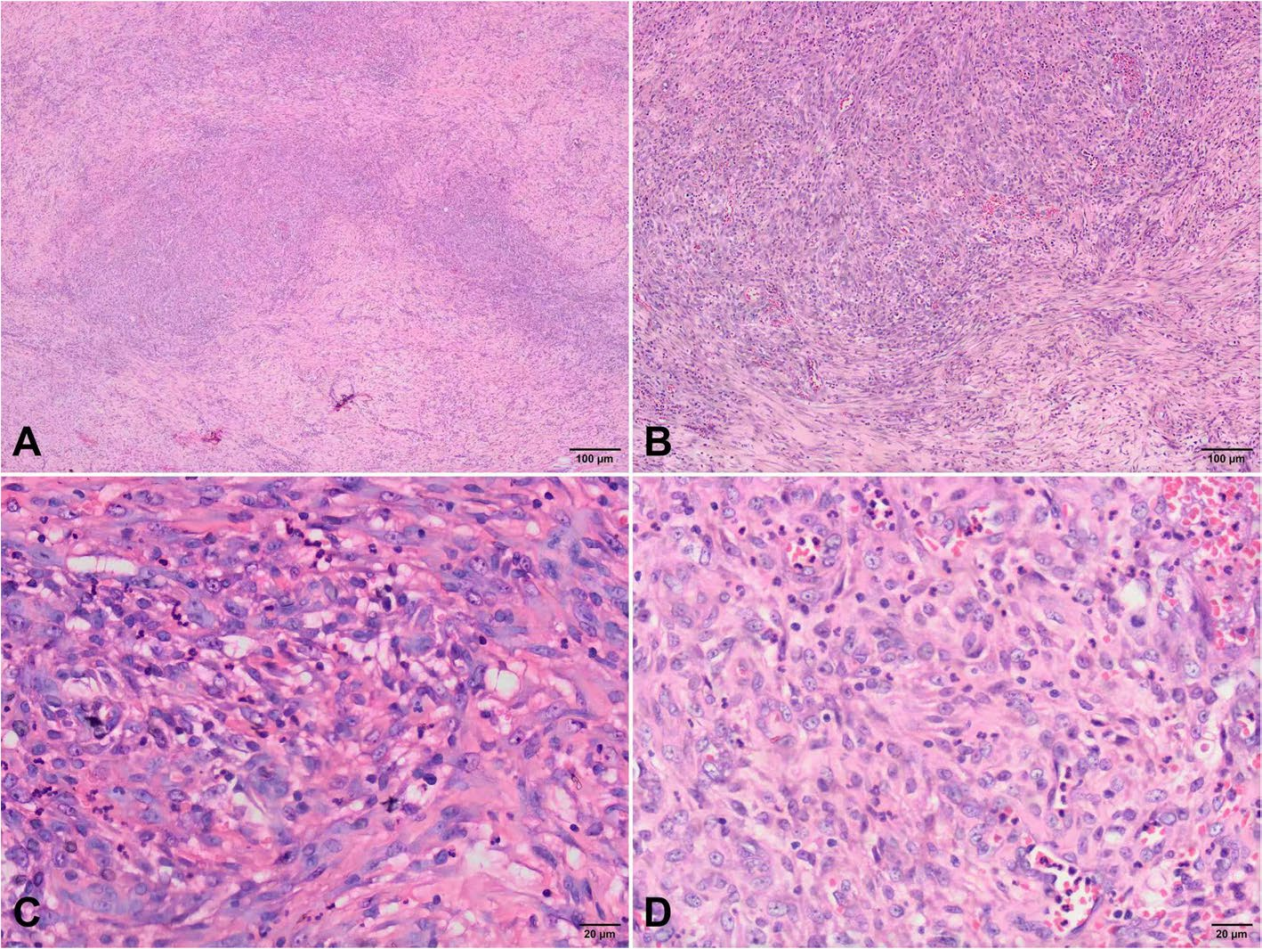
Histological characteristics of PHE tumors. **A** The tumor cells grow in nodules (× 40). **B** The tumor cells migrate with the surrounding spindle muscle epithelial cells. The tumor cells are epithelioid or fat spindle-shaped, with abundant acidophilous cytoplasm (× 100). **C** Epithelioid tumor cells are rich in eosinophilic cytoplasm, slightly heteromorphic in nucleus, with nucleolus, and mitosis in focal areas (× 400). **D** The tumor cells grow around the blood vessels. Neutrophils, plasma cells and eosinophils can be seen in the stroma (× 400)

**Fig. 2:**
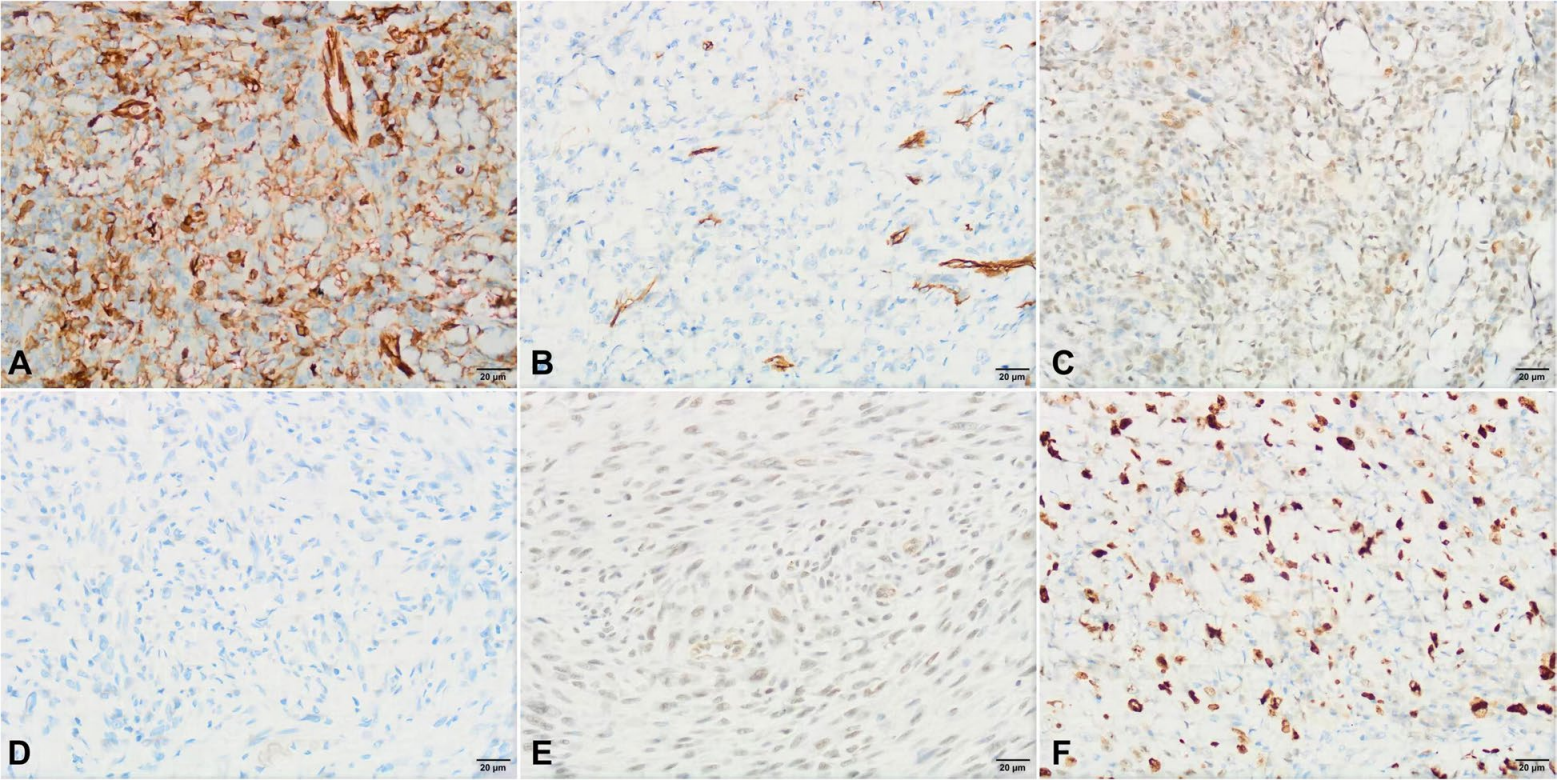
Immunohistochemical characteristics of PHE tumor cells. **A** CD31 stain: strongly positive; **B** CD34 stain: negative; **C** FLI-1 stain: positive; **D** HHV8 stain: negative; **E** INI-1 stain: retained; **F** Ki-67 stain: proliferation index 35%. (Fig. 2**A**, **D**-**F** × 400; Fig. 2**B**-**C** × 200)

Macroscopically, the 10 new tumors were 0.7–6.5 cm in diameter and were gray-white or gray-red nodules with unclear boundaries and infiltration. Some tissues were compressed and damaged to some extent. The tumors involved the dermis, subcutaneous tissues, and bones. Microscopically, various proportions of spindle cells and epithelioid cells were observed in the lesions. The spindle cells were arranged in loose strips, bundles, or nodules, and the cells were rich and plump, with eosinophilic cytoplasm and an oval nucleus. Epithelial cells were either round or pleomorphic, and some contained cytoplasmic vacuoles. In some areas, spindle cells and epithelioid endothelial cells could migrate (Fig. 1). Scattered infiltrating inflammatory cells were observed in the stroma, and mitosis was rare, without tumor necrosis or pathological mitosis. Immunohistochemical analysis revealed that cells in the 10 tumors were diffusely positive for CD31 and ERG but negative for CD34, Desmin, and S100. Some cases were positive for FLI-1, CD56, and CKpan but negative for SMA, S100, HHV8 and SOX-10. INI-1 stain is retained. The proliferation index of Ki-67 is 10–35% (Fig. 2).

### Molecular genetics

**Fig. 3:**
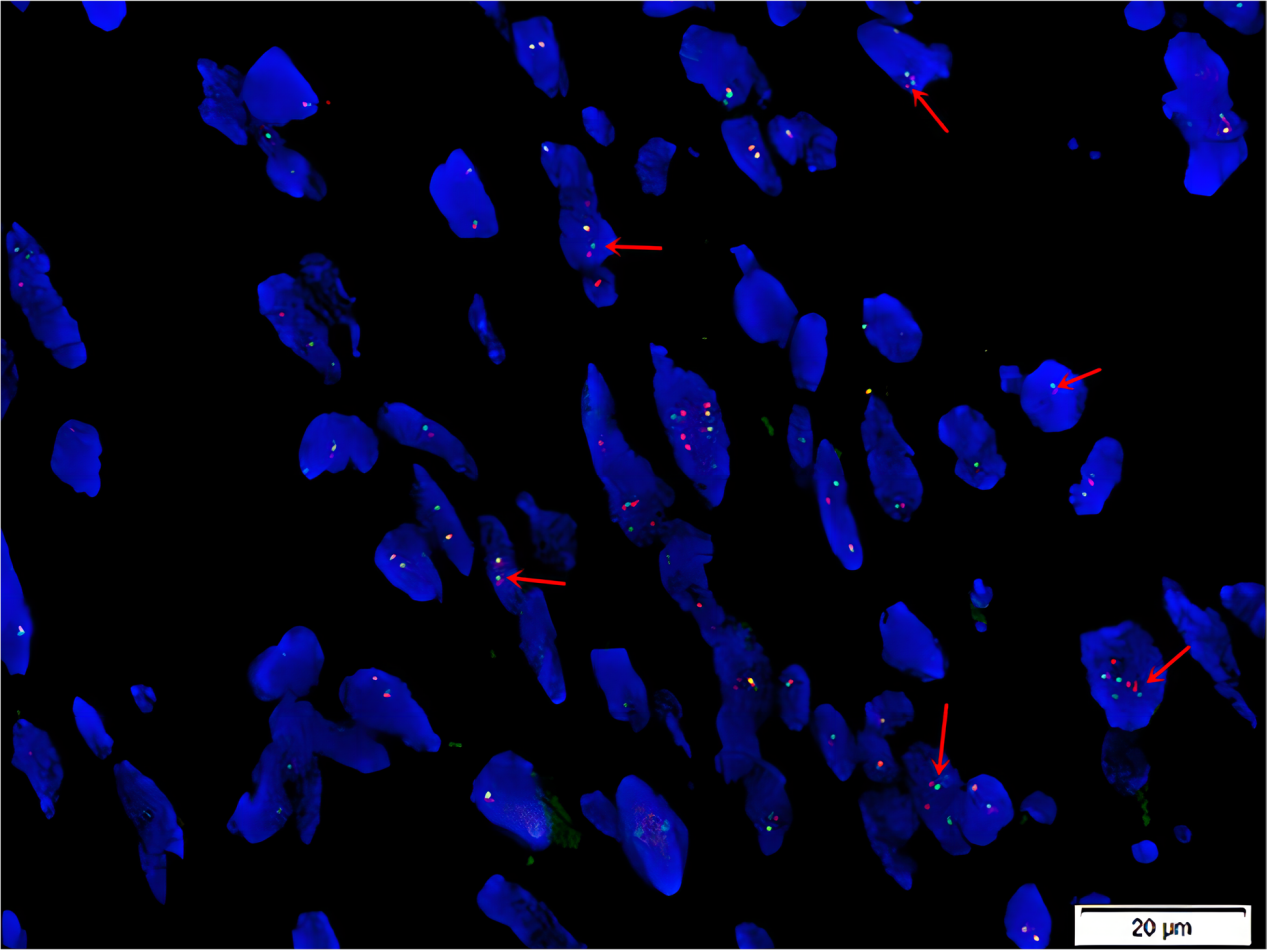
Fluorescent in situ hybridization to test FOSB breakage. Red dots: centromeres; green dots: telomeres. Red arrows indicate separation signals

Seven cases of PHE were analyzed using fluorescence in situ hybridization to detect disruptions in *FOSB* (19q13). Six cases were positive for *FOSB* breakage (Fig. 3).

### Prognosis

During 2–55 months of follow-up, four cases recurred after surgery; however, no metastasis or death occurred. Among the 175 previous cases with clinical data, 16 (9%) experienced metastasis, 41 (23%) had recurrences, and 4 died, including 2 from distant metastases, one from squamous cell carcinoma with metastasis at the base of the mouth, and one from an unknown cause. The remaining cases were cured either after initial treatment or after one or more recurrences.

## Discussion

PHE is a rare, low-grade, malignant, soft tissue tumor, officially named by the WHO in 2013, and is classified as a vascular tumor subtype [5]. Currently, less than 200 cases have been reported in the literature, primarily in case reports. PHE is common in the distal extremities of young men, especially in the lower limbs, and is often multifocal and involves multiple tissue planes. Some patients suffer from pain, and while the tumors rarely metastasize, they easily recur locally, requiring close clinical follow-up.

Macroscopically, PHE has unclear boundaries and appears as gray-white or gray-red nodules in the skin and subcutaneous tissue, with diameters ranging from several millimeters to several centimeters. Microscopically, the centers of PHE lesions are composed of plump fusiform cells and round epithelioid cells loosely arranged in sheets or bundles. The cytoplasm is eosinophilic, similar to rhabdomyoblasts. Infiltration of neutrophils, plasma cells, lymphocytes and other inflammatory cells can usually be observed in stroma. During early pathological changes, slight cell atypia is often observed; the nucleoli are small and not obvious, and mitosis is rare. In recurrent lesions, cell atypia is more obvious, with different nuclear sizes, prominent nucleoli, chromatin aggregation, and pathological mitosis [2]. Tumors often infiltrate surrounding soft tissues, such as the adipose tissue and muscle tissue. Among the 189 PHE cases reported in the past, tumors were reported in the dermis, subcutaneous tissue, muscles, and bones. Of the 50 PHE cases reported by Hornick et al*.* [1], 32 involved multiple tissue planes and the tumor edges showed infiltration. Most tumor cells were spindle cells, with a few epithelioid cells, and most tumors showed mild atypia. Billings et al*.* [3] observed intracytoplasmic vacuoles in four of seven PHE cases. Our 10 cases also involved the subcutaneous and bone tissue planes. Morphologically, fat spindle cells and rounded epithelial cells were observed, with no obvious cell atypia. Some tumor cells had a big cytoplasm and were eosinophilic, similar to rhabdomyoblasts; however, no vascular differentiation was observed. Because there is little or no obvious evidence of vascular differentiation in PHE histology, it is very difficult to diagnose the disease in this manner, and immunohistochemistry is crucial. PHE usually strongly expresses CKpan, while CD31 and ERG are expressed to different degrees. CD31, with membrane positivity and linear staining, is a characteristic immunohistochemical marker of PHE. CD34, Desmin, and S100 are not expressed, while FLI-1 is partially expressed, INI-1stain is always retained [3]. In our 10 PHE cases, CD31 and ERG were diffusely expressed, but CD34, Desmin, HHV8 and S100 were not, and some cases expressed CKpan and FLI-1, INI-1 stain is retained.

Three fusion genes have been detected in PHE: actin-beta (*ACTB*)*-FOSB*, WW domain-containing transcription regulator 1 (*WWTR1*)-*FOSB*, and serpin family E member 1 (*SERPINE1*)*-FOSB*. Among these, *ACTB-FOSB* and *SERPINE1-FOSB* are the characteristic genetic manifestations of PHE, as they are frequently detected, playing important roles in diagnosis [14]. In this group, seven patients with PHE were tested for *FOSB* cleavage and six were positive. One sample was negative; however, this may have been due to insufficient amounts of tissue for analysis.

Soft tissue tumors are widely distributed; there are many types, but all have similar structures and cell morphologies. Proper differential diagnosis is important for their treatment and therefore has clinical significance. Epithelioid sarcoma (ES) is a malignant mesenchymal tumor with an epithelioid morphology and phenotype. Apart from obvious central necrosis and more atypia, ES is very similar to PHE in clinical and histological features, and immunohistochemistry has become an important means of distinguishing between the two conditions. ES simultaneously expresses epithelial markers, such as EMA and CKpan, but does not express CD31 and FLI-1 [15]. In recent years, a lack of INI-1 expression has been identified as a specific marker for ES [16, 17], making the combined detection of INI-1, CD31, and FLI-1 helpful in distinguishing ES and PHE. Epithelial hemangioendothelioma (EH) is a central malignant vascular tumor. Unlike PHE, EH is more common in women and comprises epithelial-like endothelial cells arranged in bundles, with obvious cytoplasmic vacuolation and a stroma filled with transparent mucus. Classical EH expresses ERG, CD31, CD34, and FLI-1, while PHE usually does not express CD34. In recent years, the t(1;3)(q36;q25) chromosomal translocation, which combines the calmodulin-binding transcription activator 1 gene and *WWTR1*, has become regarded as a characteristic genetic manifestation of EH. Being a malignant tumor, the metastasis rate of EH is as high as 20–30%, and the prognosis is worse than that of PHE. Epithelioid angiosarcoma (EA) is a highly malignant tumor with obvious malignant tumor cell characteristics, such as poor differentiation, obvious atypia, and frequent mitosis. It is prone to local recurrence and metastasis in a short time. Compared to PHE, the tumor cell atypia of EA is more obvious, the nuclear division is more common, and tumor tissue often bleed and die. In most cases, anastomotic vascular cavities can be seen. Regarding immunohistochemistry, unlike in PHE, CD31 and FLI-1 are usually negative in EA, while CD34 is partially positive.

PHE is a type of vascular tumor that easily recurs locally but rarely metastasizes. Most patients with PHE to date have only required tumor resection, while a few underwent extensive resection or even amputation, along with adjuvant radiotherapy and chemotherapy. All 10 of our cases were resected, and two also received chemotherapy. Among 173 PHE patients with clinical data, 138 (80%) underwent surgical resection and 15 (8.67%) were treated with adjuvant radiotherapy, chemotherapy, or immunosuppressants. In recent years, increasing attention has been paid to the use of mechanistic target of rapamycin kinase (mTOR) inhibitors in PHE treatment [18–21]. mTOR activity is inhibited by rapamycin, which exerts antitumor effects by inhibiting proliferation and cell cycle progression. For patients with PHE who experience either adverse effects after radiotherapy and chemotherapy or metastasis, clinical symptoms improve after treatment with mTOR inhibitors. David et al*.* [22] studied the effect of the SERPINE1-FOSB fusion protein on endothelial cell function and its interaction with telatinib and suggested that telatinib may be a highly specific targeted treatment option for patients with PHE who are ineligible for surgery. Davis et al*.* [23] described a multifocal case of PHE involving both bone and soft tissues and proposed a treatment strategy using radiofrequency ablation and extensive resection for the bone and soft tissue lesions, respectively. These new therapeutic strategies provide new PHE treatment options; however, they are all case reports and will require verification with more cases.

In conclusion, PHE is a rare intermediate soft-tissue tumor with inert biological behavior, which often recurs but only occasionally metastasizes. Because of its lack of specific morphological features, it is easily misdiagnosed based solely on histomorphology. However, PHE exhibits characteristic immunohistochemical markers and molecular changes, which play extremely important roles in its diagnosis. To date, less than 200 cases of PHE have been reported worldwide. As more cases are reported in the future, our understanding of PHE will deepen.

## Supplementary Information

**Additional file 1: Supplementary Table 1.** Clinical data from 180 previously published PHE cases.
