# Supplementary material for: Clinicopathological study of pseudomyogenic hemangioendothelioma
Source: Diagn Pathol. 2023 Feb 20;18:25. doi: 10.1186/s13000-023-01309-9 (PMC9940391; doi:10.1186/s13000-023-01309-9)
Supplement: Supplementary file 1 — Additional file 1: Supplementary Table 1. Clinical data from 180 previously published PHE cases. [file 13000_2023_1309_MOESM1_ESM.docx]

**Supplementary Table 1. Clinical data from 180 previously published PHE cases**

| **Author** | **Cases** | **Sex (M:F)** | **Age (average)** | **Site** | **Sin/Mul** | **Recurrence/Metastasis** | **Treatment** | **Prognostic status** |
| --- | --- | --- | --- | --- | --- | --- | --- | --- |
| Mirra et al., 1992 [1] | 5 | 4:1 | 25 | 4 LL, 1 UL | 4 Mul,1 Sin | 1 Met | 2 E, 2 E + C, 1 E + R + C | 4 NED, 1 DOD |
| Billings et al., 2003 [2] | 7 | 4:3 | 30 | 4 LL, 1 UL, 1 T, 1 HN | 6 Sin,1 Mul | 2 Rec,1 Met | E + R | 2 AWD, 5 NED |
| Tokyol et al., 2005 [3] | 1 | F | 70 | UL (BI) | Mu | NO | E + R | NED |
| Watabe et al., 2009 [4] | 1 | M | 56 | \| UL \| \| --- \| | Mul | Rec | E | NA |
| Hornick et al., 2011 [5] | 50 | 41:9 | 31 | 54% LL,24% UL, 18% T,4% HN | 66% Mul | 18 Rec, 2 Met | 46 E, 8 E + R, 6 E + C | 27 NED, 2 AWD, 1 DOD, 20 NA |
| Trombetta et al., 2011 [6] | 1 | F | 14 | T | Mul | Rec | E | NED |
| Cai et al., 2011 [7] | 3 | 3:0 | 49 | Neck, iliac region, bilateral neck, and shoulder region | Mul | 1 Rec, 1 Met | E | 2 SWD, 1 AWD |
| McGinity et al., 2012 [8] | 1 | M | 25 | T (BI) | Sin | NA | E | NA |
| Amary et al., 2012 [9] | 5 | 3:2 | 36 | 4 LL, 1 UL | 4 Mul,1 Sin | 1 Met | 3 E, 1 C | 3 AWD, 2 SWD |
| Sheng et al., 2013 [10] | 1 | F | 10 | LL (BI) | Mul | Rec | E | DP |
| \| Sheng et al., 2013 [11] \| \| --- \| | 1 | M | 22 | LL, T | Mul | Met | E + C | Survival |
| \| Walther et al., 2013 [12] \| \| --- \| | 1 | M | 16 | LL | Sin | Rec | Curettage | DP |
| \| Stuart et al., 2013 [13] \| \| --- \| | 1 | M | 30 | LL | Mu | NA | C | DP |
| \| Requena et al., 2013 [14] \| \| --- \| | 2 | 1:1 | 23 | 1 LL, 1 HN | Mul | NO | E | NED |
| Karakasli et al., 2014 [15] | 1 | M | 54 | UL (BI) | Sin | Rec | E | NA |
| \| Righi et al., 2014 [16] \| \| --- \| | 2 | 1:1 | 46 | 1 LL, 1 UL | Mul | 1 Rec, 1 NA | E | Survival |
| \| bryanton et al., 2015 [17] \| \| --- \| | 1 | M | 59 | T, HN (BI) | Mu | NA | Conservative treatment | NA |
| \| Joseph et al., 2015 [18] \| \| --- \| | 2 | 2:0 | 33 | RL, ilium | 1 Mul | 1 Met | E + C | 2 AWD |
| \| Ide et al., 2015 [19] \| \| --- \| | 1 | M | 43 | Penis | Mul | NO | E | NED |
| \| Shah et al., 2015 [20] \| \| --- \| | 1 | M | 86 | LL (BI) | Mul | Met | Palliative treatment | DOD |
| \| Fan et al., 2015 [21] \| \| --- \| | 1 | F | 49 | LL | Sin | Rec + Met | E | AWD |
| \| Davis et al., 2015 [22] \| \| --- \| | 1 | M | 20 | LL | Mul | NO | R | NED |
| \| Rawal et al., 2016 [23] \| \| --- \| | 1 | F | 21 | OR | Sin | NO | E | NED |
| \| Tsubokawa et al., 2016 [24] \| \| --- \| | 1 | M | 68 | T | Sin | NO | E | NED |
| \| Sugita et al., 2016 [25] \| \| --- \| | 4 | 2:2 | 31 | BI,LL | Mul | NA | NA | NA |
| \| Inyang et al., 2016 [26] \| \| --- \| | 10 | 9:1 | 37 | 45% LL, 25% T (BI),15% UL | Mul | 4 Rec, 2 Met | 5 Biopsy, 4 E, 1 R | 5 AWD, 1 OD, 1 NED |
| \| Ye et al., 2016 [27] \| \| --- \| | 1 | F | 14 | LL | Mul | Rec | E | NED |
| \| Rekhi et al., 2016 [28] \| \| --- \| | 1 | M | 17 | \| LL \| \| --- \| | Mul | NO | NA | NED |
| \| Cheo et al., 2017 [29] \| \| --- \| | 1 | M | 15 | \| LL,T \| \| --- \| | Mul | NA | E | NA |
| \| Alegría-Landa et al., 2017 [30] \| \| --- \| | 1 | F | 25 | LL | Mul | NA | NA | NA |
| \| Gabor et al., 2017 [31] \| \| --- \| | 1 | M | 9 | LL (BI) | Mul | Rec | \| MTOR Inhibitor \| \| --- \| | AWD |
| \| Ozeki et al., 2017 [32] \| \| --- \| | 1 | M | 15 | \| LL, T (BI) \| \| --- \| | Mul | NA | \| MTOR Inhibitor \| \| --- \| | AWD |
| \| Agaram et al., 2018 [33] \| \| --- \| | 15 | 11:4 | 35 | 8 LL, 5 T, 2 UL | 10 Mul, 5 Sin | NO | 10 E, 1 E + C + R, 4 NA | 7 NED, 2 AWD |
| \| Pradhan et al., 2018 [34] \| \| --- \| | 8 | 7:1 | 29 | 6 LL,2 UL | 5 Mul | 1 Rec,2 NA | 6 E, 2 NA | NED |
| \| Squillaci et al., 2018 [35] \| \| --- \| | 1 | F | 46 | \| LL \| \| --- \| | NA | NA | NA |  |
| \| Pranteda et al., 2018 [36] \| \| --- \| | 1 | M | 17 | LL | Mul | NO | Conservative treatment | AWD |
| \| Raftopoulos et al., 2018 [37] \| \| --- \| | 1 | M | 65 | LL | Mul | NO | E | NED |
| \| van Ijzendoorn et al., 2018 [38] \| \| --- \| | 1 | M | 17 | HN | Mul | Met | Inhibitor | NED |
| \| Panagopoulos et al., 2019 [39] \| \| --- \| | 1 | F | 33 | T (BI) | Sin | NO | NA | NA |
| \| Sun et al., 2019 [40] \| \| --- \| | 24 | 18:6 | 34 | 63% LL, 25% T, 8% UL, 4% HN | 16 Mul | 4 Rec,1 Met | 1 curettage, 21 E | 11 AWD, 6 SWD, 7 NA |
| \| Sun et al., 2019 [41] \| \| --- \| | 1 | F | 51 | Vulva | Mul | NO | E | NED |
| \| Ge et al., 2019 [42] \| \| --- \| | 1 | F | 43 | Mammary gland | Mul | NO | E | NED |
| \| Bartholomew et al., 2019 [43] \| \| --- \| | 1 | M | 19 | LL | Mul | Met | E + mTOR Inhibitor | NA |
| \| Ansai et al., 2019 [44] \| \| --- \| | 1 | F | 28 | LL | Sin | NO | E | NED |
| \| Otani et al., 2019 [45] \| \| --- \| | 1 | F | 20 | LL (BI) | Mul | NO | C | AWD |
| \| Dianat et al., 2019 [46] \| \| --- \| | 1 | M | 63 | T (BI) | Mul | NO | E | NED |
| \| Kosemehmetoglu et al., 2019 [47] \| \| --- \| | 5 | 4:1 | 28 | 4 LL (BI), 1 T (BI) | 4 Mul | NO | 1 E | NED |
| Danforth et al., 2019 [48] | 1 | M | 6 | \| LL (BI) \| \| --- \| | Mul | NO | Drugs | AWD |
| \| Xia et al., 2020 [49] \| \| --- \| | 1 | M | 34 | HN (BI) | Mul | NA | E | NED |
| \| Shackelford et al., 2020 [50] \| \| --- \| | 1 | F | 33 | Oral | Sin | NO | E | NED |
| Wei et al., 2020 [51] | 2 | 1:1 | 40 | LL | Mul | 2 Rec | E | NA |

**Abbreviations:** UL, upper limb; LL, lower limb; T, trunk; HN, head and neck; BI, bone involvement; Met, metastasis, Rec, recurrence; E, excision; C, chemotherapy; R, radiotherapy; Mul, multifocal; Sin, single focus; NA, not available; mTOR, mechanistic target of rapamycin kinase; NED, no evidence of disease; AWD, alive with disease; DP, disease progression; DOD, death of disease.

**References**

[1] J.M. Mirra, S. Kessler, S. Bhuta, J. Eckardt, The fibroma-like variant of epithelioid sarcoma. A fibrohistiocytic/myoid cell lesion often confused with benign and malignant spindle cell tumors. Cancer 69 (1992) 1382-1395. doi: 10.1002/1097-0142(19920315)69:6<1382::aid-cncr2820690614>3.0.co;2-y.

[2] S.D. Billings, A.L. Folpe, S.W. Weiss, Epithelioid sarcoma-like hemangioendothelioma. Am J Surg Pathol 27 (2003) 48-57. doi: 10.1097/00000478-200301000-00006.

[3] C. Tokyol, N. Uzum, I. Kuru, O. Uluoglu, Epithelioid sarcoma-like hemangioendothelioma: a case report. Tumori 91 (2005) 436-439.

[4] A. Watabe, R. Okuyama, A. Hashimoto, M. Hosaka, M. Hatori, Y. Kariya, M. Watanabe, H. Hashimoto, H. Tagami, S. Aiba, Epithelioid sarcoma-like haemangioendothelioma: a case report. Acta Derm Venereol 89 (2009) 208-209. doi: 10.2340/00015555-0599.

[5] J.L. Hornick, C.D. Fletcher, Pseudomyogenic hemangioendothelioma: a distinctive, often multicentric tumor with indolent behavior. Am J Surg Pathol 35 (2011) 190-201. doi: 10.1097/PAS.0b013e3181ff0901.

[6] D. Trombetta, L. Magnusson, F.V. von Steyern, J.L. Hornick, C.D. Fletcher, F. Mertens, Translocation t(7;19)(q22;q13)-a recurrent chromosome aberration in pseudomyogenic hemangioendothelioma? Cancer Genet 204 (2011) 211-215. doi: 10.1016/j.cancergen.2011.01.002.

[7] J.N. Cai, F. Peng, L.X. Li, Y.F. Cheng, J. Wang, [Epithelioid sarcoma-like hemangioendothelioma: a clinicopathologic and immunohistochemical study of 3 cases]. Zhonghua Bing Li Xue Za Zhi 40 (2011) 27-31. Chinese.

[8] M. McGinity, V. Bartanusz, B. Dengler, L. Birnbaum, J. Henry, Pseudomyogenic hemangioendothelioma (epithelioid sarcoma-like hemangioendothelioma, fibroma-like variant of epithelioid sarcoma) of the thoracic spine. Eur Spine J 22 Suppl 3 (2013) S506-S511. doi: 10.1007/s00586-013-2727-3.

[9] M.F. Amary, P. O'Donnell, F. Berisha, R. Tirabosco, T. Briggs, R. Pollock, A.M. Flanagan, Pseudomyogenic (epithelioid sarcoma-like) hemangioendothelioma: characterization of five cases. Skeletal Radiol 42 (2013) 947-957. doi: 10.1007/s00256-013-1577-8.

[10] W.Q. Sheng, J. Wang, Primary pseudomyogenic haemangioendothelioma of bone. Histopathology 61 (2012) 1219-1224. doi: 10.1111/j.1365-2559.2012.04347.x.

[11] W. Sheng, Y. Pan, J. Wang, Pseudomyogenic hemangioendothelioma: report of an additional case with aggressive clinical course. Am J Dermatopathol 35 (2013) 597-600. doi: 10.1097/DAD.0b013e31827c8051.

[12] C. Walther, J. Tayebwa, H. Lilljebjorn, L. Magnusson, J. Nilsson, F.V. von Steyern, I. Ora, H.A. Domanski, T. Fioretos, K.H. Nord, C.D. Fletcher, F. Mertens, A novel SERPINE1-FOSB fusion gene results in transcriptional up-regulation of FOSB in pseudomyogenic haemangioendothelioma. J Pathol 232 (2014) 534-540. doi: 10.1002/path.4322.

[13] L.N. Stuart, J.M. Gardner, S.R. Lauer, D.K. Monson, D.C. Parker, M.A. Edgar, Epithelioid sarcoma-like (pseudomyogenic) hemangioendothelioma, clinically mimicking dermatofibroma, diagnosed by skin biopsy in a 30-year-old man. J Cutan Pathol 40 (2013) 909-913. doi: 10.1111/cup.12196.

[14] L. Requena, C. Santonja, J.L. Martinez-Amo, C. Saus, H. Kutzner, Cutaneous epithelioid sarcomalike (pseudomyogenic) hemangioendothelioma: a little-known low-grade cutaneous vascular neoplasm. JAMA Dermatol 149 (2013) 459-465. doi: 10.1001/jamadermatol.2013.3190.

[15] A. Karakasli, A. Karaaslan, M. Erduran, S. Capkin, E.B. Tuna, H. Havitcioglu, Pseudomyogenic (Epithelioid sarcoma-like) hemangioendothelioma with bone invasion. J Orthop 11 (2014) 197-199. doi: 10.1016/j.jor.2014.06.019.

[16] A. Righi, M. Gambarotti, P. Picci, A.P. Dei Tos, D. Vanel, Primary pseudomyogenic haemangioendothelioma of bone: report of two cases. Skeletal Radiol 44 (2015) 727-731. doi: 10.1007/s00256-014-2024-1.

[17] M. Bryanton, W. Makis, Pseudomyogenic Hemangioendothelioma Mimicking Multiple Myeloma on 18F-FDG PET/CT, Followed by Spontaneous Regression. Clin Nucl Med 40 (2015) 579-581. doi: 10.1097/RLU.0000000000000800.

[18] J. Joseph, W.L. Wang, M. Patnana, N. Ramesh, R. Benjamin, S. Patel, V. Ravi, Cytotoxic and targeted therapy for treatment of pseudomyogenic hemangioendothelioma. Clin Sarcoma Res 5 (2015) 22-27. doi: 10.1186/s13569-015-0037-8.

[19] Y.H. Ide, Y. Tsukamoto, T. Ito, T. Watanabe, N. Nakagawa, T. Haneda, M. Nagai, K. Yamanishi, S. Hirota, Penile pseudomyogenic hemangioendothelioma/epithelioid sarcoma-like hemangioendothelioma with a novel pattern of SERPINE1-FOSB fusion detected by RT-PCR--report of a case. Pathol Res Pract 211 (2015) 415-420. doi: 10.1016/j.prp.2015.02.003.

[20] Shah AR, Fernando M, Musson R, Kotnis N. An aggressive case of pseudomyogenic haemangioendothelioma of bone with pathological fracture and rapidly progressive pulmonary metastatic disease: case report and review of the literature. Skelet Radiol 2015; 44: 1381-1386.

[21] C. Fan, L. Yang, X. Lin, E. Wang, Pseudomyogenic hemangioendothelioma/epithelioid sarcoma-like hemangioendothelioma of the lower limb: report of a rare case. Diagn Pathol 10 (2015) 150. doi: 10.1186/s13000-015-0384-z.

[22] A.T. Davis, A.M. Guo, N.J. Phillips, D.D. Greenberg, A novel treatment for bone lesions of multifocal epithelioid sarcoma-like hemangioendothelioma. Skeletal Radiol 44 (2015) 1013-1019. doi: 10.1007/s00256-014-2089-x.

[23] Rawal YB, Anderson KM, Dodson TB. Pseudomyogenic hemangioendothelioma: A vascular tumor previously undescribed in the oral cavity. Head Neck Pathol 2017; 11: 525-530.

[24] N. Tsubokawa, H. Harada, D. Taniyama, T. Uemura, K. Kuraoka, Y. Yamashita, Epithelioid sarcoma-like hemangioendothelioma on the chest wall. Asian Cardiovasc Thorac Ann 24 (2016) 814-817. doi: 10.1177/0218492316664672.

[25] S. Sugita, H. Hirano, N. Kikuchi, T. Kubo, H. Asanuma, T. Aoyama, M. Emori, T. Hasegawa, Diagnostic utility of FOSB immunohistochemistry in pseudomyogenic hemangioendothelioma and its histological mimics. Diagn Pathol 11 (2016) 75. doi: 10.1186/s13000-016-0530-2.

[26] A. Inyang, F. Mertens, F. Puls, V. Sumathi, C. Inwards, A. Folpe, C.H. Lee, Y. Zhang, P. Symmans, B. Rubin, G.P. Nielsen, V.H. Nguyen, A.E. Rosenberg, Primary Pseudomyogenic Hemangioendothelioma of Bone. Am J Surg Pathol 40 (2016) 587-598. doi: 10.1097/PAS.0000000000000613.

[27] C. Ye, X. Yu, J. Zeng, H. Liu, M. Dai, Pseudomyogenic hemangioendothelioma secondary to fibrous dysplasia of the left lower extremity in a 14-year-old female: a case report. World J Surg Oncol 14 (2016) 198. doi: 10.1186/s12957-016-0955-9.

[28] B. Rekhi, A. Gulia, V. Rangarajan, A rare case of multifocal pseudomyogenic hemangioendothelioma, involving soft tissues and bone, misdiagnosed as a rhabdomyosarcoma: Diagnostic and treatment implications. Indian J Pathol Microbiol 59 (2016) 382-385. doi: 10.4103/0377-4929.188144.

[29] F.F. Cheo, K. Sittampalam, Pseudomyogenic (epithelioid sarcoma-like) hemangioendothelioma - a rare vascular neoplasm with deceptive morphology and distinctive immunophenotype. Malays J Pathol 39 (2017) 305-309.

[30] V. Alegria-Landa, C. Santonja, M. Jo-Velasco, H. Kutzner, L. Requena, Cutaneous pseudomyogenic (epithelioid sarcoma-like) haemangioendothelioma FOSB immunohistochemistry demonstrating the SERPINE1-FOSB fusion gene. J Eur Acad Dermatol Venereol 31 (2017) e550-e552. doi: 10.1111/jdv.14417.

[31] K.M. Gabor, Z. Sapi, L.G. Tiszlavicz, A. Fige, C. Bereczki, K. Bartyik, Sirolimus therapy in the treatment of pseudomyogenic hemangioendothelioma. Pediatr Blood Cancer 65 (2018) e26781-e26784. doi: 10.1002/pbc.26781.

[32] M. Ozeki, A. Nozawa, K. Kanda, T. Hori, A. Nagano, A. Shimada, T. Miyazaki, T. Fukao, Everolimus for Treatment of Pseudomyogenic Hemangioendothelioma. J Pediatr Hematol Oncol 39 (2017) e328-e331. doi: 10.1097/MPH.0000000000000778.

[33] N.P. Agaram, L. Zhang, P. Cotzia, C.R. Antonescu, Expanding the Spectrum of Genetic Alterations in Pseudomyogenic Hemangioendothelioma With Recurrent Novel ACTB-FOSB Gene Fusions. Am J Surg Pathol 42 (2018) 1653-1661. doi: 10.1097/PAS.0000000000001147.

[34] D. Pradhan, K. Schoedel, R.L. McGough, S. Ranganathan, U.N.M. Rao, Pseudomyogenic hemangioendothelioma of skin, bone and soft tissue-a clinicopathological, immunohistochemical, and fluorescence in situ hybridization study. Hum Pathol 71 (2018) 126-134. doi: 10.1016/j.humpath.2017.10.023.

[35] S. Squillaci, A. Pitino, C. Spairani, P.C. Rassu, E. Chiapuzzo, H. Kutzner, Primary pseudomyogenic hemangioendothelioma of bone: case report and review of the literature. Pathologica 110 (2018) 96-101.

[36] G. Pranteda, F. Magri, M. Muscianese, F. Pigliacelli, A. D'Arino, A. Federico, G. Pranteda, A. Bartolazzi, The management of pseudomyogenic hemangioendothelioma of the foot: A case report and review of the literature. Dermatol Ther 31 (2018) e12725. doi: 10.1111/dth.12725.

[37] E. Raftopoulos, M. Royer, M. Warren, J. Zhao, W. Rush, Pseudomyogenic Hemangioendothelioma: Case Report and Review of the Literature. Am J Dermatopathol 40 (2018) 597-601. doi: 10.1097/DAD.0000000000001104.

[38] I.D.G.P. van, S. Sleijfer, H. Gelderblom, F. Eskens, G. van Leenders, K. Szuhai, J. Bovee, Telatinib Is an Effective Targeted Therapy for Pseudomyogenic Hemangioendothelioma. Clin Cancer Res 24 (2018) 2678-2687. doi: 10.1158/1078-0432.CCR-17-3512.

[39] I. Panagopoulos, I. Lobmaier, L. Gorunova, S. Heim, Fusion of the Genes WWTR1 and FOSB in Pseudomyogenic Hemangioendothelioma. Cancer Genomics Proteomics 16 (2019) 293-298. doi: 10.21873/cgp.20134.

[40] Y. Sun, M. Zhao, I.W. Lao, L. Yu, J. Wang, The clinicopathological spectrum of pseudomyogenic hemangioendothelioma: report of an additional series with review of the literature. Virchows Arch 477 (2020) 231-240. doi: 10.1007/s00428-020-02753-4.

[41] Y.F. Sun, J. Wang, Primary pseudomyogenic hemangioendothelioma of the vulva: a rare location for a rare entity. Diagn Pathol 14 (2019) 66-71. doi: 10.1186/s13000-019-0846-9.

[42] Y. Ge, X. Lin, F. Zhang, F. Xu, L. Luo, W. Huang, Z. Liu, Y. Liu, Z. Li, A rare case of pseudomyogenic hemangioendothelioma (PHE)/epithelioid sarcoma-like hemangioendothelioma (ES-H) of the breast first misdiagnosed as metaplastic carcinoma by FNAB and review of the literature. Diagn Pathol 14 (2019) 79-85. doi: 10.1186/s13000-019-0857-6.

[43] J.R. Bartholomew, M. Tran, Images in Vascular Medicine. Pseudomyogenic hemangioendothelioma - A rare vascular tumor. Vasc Med 25 (2020) 93-94. doi: 10.1177/1358863X19888683.

[44] S.I. Ansai, M. Morimoto, S. Akaishi, Pseudomyogenic Hemangioendothelioma. J Nippon Med Sch 86 (2019) 126-130. doi: 10.1272/jnms.JNMS.2019_86-209.

[45] S. Otani, R. Nakayama, T. Sekita, T. Hirozane, N. Asano, K. Nishimoto, A. Sasaki, H. Okita, H. Morioka, M. Nakamura, M. Matsumoto, Pseudomyogenic hemangioendothelioma of bone treated with denosumab: a case report. BMC Cancer 19 (2019) 872. doi: 10.1186/s12885-019-6072-8.

[46] S. Dianat, H. Yousaf, P. Murugan, S. Marette, Pseudomyogenic hemangioendothelioma-A case report and review of the literature. Radiol Case Rep 14 (2019) 1228-1232.  doi: 10.1016/j.radcr.2019.06.029.

[47] K. Kosemehmetoglu, B. Rekhi, P.E. Wakely, Jr., V. Pant, S. Dervisoglu, U. Aydingoz, Pseudomyogenic (epithelioid sarcoma-like) hemangioendothelioma of bone: Clinicopathologic features of 5 cases. Ann Diagn Pathol 41 (2019) 116-123. doi: 10.1016/j.anndiagpath.2019.06.003.

[48] O.M. Danforth, K. Tamulonis, K. Vavra, C. Oh, A. Brickman, J. Ebersole, J. Cameron, B. Mahon, P. Kent, Effective Use of Sirolimus and Zoledronic Acid for Multiosteotic Pseudomyogenic Hemangioendothelioma of the Bone in a Child: Case Report and Review of Literature. J Pediatr Hematol Oncol 41 (2019) 382-387. doi: 10.1097/MPH.0000000000001459.

[49] R.H. Xia, L. Zhu, L.Z. Wang, Z. Tian, C.Y. Zhang, Y.H. Hu, T. Gu, J. Li, Primary pseudomyogenic hemangioendothelioma of right maxilla: a case with immunohistochemistry and FOSB rearrangement study. Oral Surg Oral Med Oral Pathol Oral Radiol 130 (2020) e96-e105. doi: 10.1016/j.oooo.2019.12.013.

[50] A.J. Shackelford, C.R. Canterbury, M.A. Perrino, J. Wang, E.M. Philipone, S.M. Peters, Oral Pseudomyogenic Hemangioendothelioma: Case Report and Review of the Literature. Head Neck Pathol 14 (2020) 1134-1138. doi: 10.1007/s12105-020-01137-z.

[51] Wei, Z. Liao, G. Zhao, N. Nahar, C. Zhang, J. Lu, Y. Yang, J. Yang, Clinicopathological features of pseudomyogenic hemangioendothelioma and precision therapy based on whole exome sequencing. Cancer Commun (Lond) 40 (2020) 197-201. doi: 10.1002/cac2.12020.
